# The knowledge, attitudes and behaviors of hospital nurses on smoking cessation interventions: a cross-sectional study

**DOI:** 10.1186/s12912-023-01394-7

**Published:** 2023-07-03

**Authors:** Marta Čivljak, Lovro Ačkar, Livia Puljak

**Affiliations:** Center for Evidence-Based Medicine and Health Care, Catholic University of Croatia, Ilica 242, Zagreb, 10000 Croatia

**Keywords:** Tobacco smoking, Nurses, Smoking cessation, Interventions, Education

## Abstract

**Background:**

Smoking is a major public health problem in Croatia. It is unknown to what extent nurses in Croatia use interventions for smoking cessation to help their patients. This study aimed to analyze the knowledge, attitudes and behaviors of hospital nurses on smoking cessation interventions.

**Methods:**

We conducted a cross-sectional study in Zagreb, Croatia, in 2022 on a convenient sample of hospital nurses. We collected data with a questionnaire that included sociodemographic questions and questions about the frequency of implementation of 5 A’s (Ask, Advise, Assess, Assist, Arrange) interventions for smoking cessation during their work using the Helping Smokers Quit (HSQ) survey, participants’ attitudes and knowledge about smoking cessation skills and the smoking status of the nurses.

**Results:**

There were 824 nurses employed in the targeted departments; 258 nurses participated in the study (response rate: 31%). Among them, 43% responded that they always ask patients about their use of tobacco products. Only 2.7% indicated that they always help the patient to stop smoking. Very few (2%) attended any training in the past two years about helping patients to quit smoking, and 82% never had such training. 44% of the included nurses were smokers. Nurses who smoked stated more frequently than nonsmokers they should not be role models for their patients by avoiding smoking (P 0.001). Additionally, patients were less frequently questioned about their inability to stop smoking by nurses who smoked than nurses who did not smoke (P = 0.010).

**Conclusion:**

Even though smoking cessation interventions delivered by nurses were proven effective, such interventions are used by a small number of surveyed nurses. A small number of nurses have received training to help them support smokers in quitting. The high smoking prevalence among nurses may impact their attitudes and the implementation of workplace smoking cessation efforts.

**Supplementary Information:**

The online version contains supplementary material available at 10.1186/s12912-023-01394-7.

## Background

Smoking is a global public health problem, with 1.3 billion smokers worldwide [1]. It is estimated that 8 million people die annually from the effects of smoking globally, of which 1.2 million deaths are associated with passive smoking [1].

Smoking is also a public health problem in Croatia, as evidenced by the results of the study conducted in 2014 and 2015 by the relevant Croatian and international public health authorities. According to the study results, 31% of the population aged over 15 smoked in Croatia (35.3% of men and 27.1% of women) [2].

The World Health Organization (WHO) Framework Convention on Tobacco Control (FCTC) was developed in response to the globalization of the tobacco epidemic. The FCTC is the first international global health treaty and the evidence-based treaty that reaffirms the right of all people to the highest standard of health, which came into force in 2005 [3]. The WHO introduced the six MPOWER measures in 2008 to guide countries in this process, each corresponding to one or more FCTC provisions. The MPOWER package includes: monitoring tobacco use and tobacco control measures; protecting people from tobacco smoke; offering help (e.g., treatments) to quit tobacco; warning people about the dangers of tobacco; enforcing bans on tobacco advertising, promotion and sponsorship and raising tobacco taxes [4].

Although Croatia is one of the countries that are a Party to the WHO FCTC, comprehensive tobacco control policies are yet to be fully implemented. The least implemented FCTC article is cessation support through advice from healthcare providers, telephone quit lines and easily-accessible or low-cost medications (Article 14 of the FCTC) [5].

Nurses make up the largest healthcare profession in the world. Approximately 27 million men and women comprise the global nursing and midwifery workforce, accounting for nearly 50% of the global health workforce [6]. In addition, there is evidence that behavioral support to motivate and sustain smoking cessation delivered by nurses can increase the number of people who achieve prolonged abstinence [7].

Nurses play a vital role in primary health care delivery worldwide – including research, disease prevention, treating the injured, palliative care and more – which is represented through several World Health Assembly resolutions [6]. Among other roles, Croatian nursing law stipulates that a nurse has to be a counsellor to help people focus on a goal or outcome, develop strategies that support self-care, and enable individuals and their families to take responsibility for and participate in decisions about their health [8]. Thus, advising patients to stop smoking is part of a nurse’s role.

Two well-known methods to help people quit smoking are the ABC method (ask, brief advice, cessation support) [9], and the 5As (ask for the smoking status, brief advice to quit, assess the motivation to quit, assist by providing evidence-based treatment, arrange follow-up) [10].

Furthermore, the counseling intervention can be captured by the 5Rs: relevance, risks, rewards, roadblocks, and repetition, which includes an additional brief intervention to enhance the motivation to quit in unmotivated smokers [10].

There is evidence that increasing the reach and intensity of smoking cessation services across the health sector might help people quit smoking. For example, a Cochrane systematic review published by Rigotti et al. in 2012 examined interventions offered to hospitalized patients for smoking cessation, including behavioral, pharmacological or multicomponent interventions. They concluded that intensive interventions that begin in the hospital with nicotine replacement therapy increase the rate of smoking cessation [11]. Additionally, multiple studies demonstrated that interventions provided by nurses could lead to an increase in the number of people who stop smoking or prolong abstinence [7, 12].

In 2015, Sarna et al. conducted a study in the Czech Republic and examined the frequency of nurses’ interventions in helping smokers to quit smoking, their attitudes and skills, and investigated the association between nurses’ smoking status and the level of intervention they implement [13]. The study showed that consistent intervention by nurses to help patients quit smoking was relatively rare in the Czech Republic, and some nurses were unwilling to intervene with patients on the topic of smoking cessation. Although the study of Sarna et al. included only nurses in the Czech Republic, it is possible that these results may be similar for nurses in other countries of Eastern and Southeastern Europe since smoking rates are very high in the countries of that area (both in the population and among nurses), and interventions by nurses (and other professions) are not part of routine clinical practice [13].

Nurses have an important window of opportunity to intervene with patients during their hospital stay or introduce the notion of not resuming smoking upon hospital discharge [7]. In addition, previous research shows that training nurses to implement smoking cessation interventions is extremely important and increases their willingness to participate in such programs [14, 15].

Li et al. have summarized literature about factors associated with nursing interventions for smoking cessation. They reported that nurses’ attitudes or perceptions, social influence, organizational factors and self-efficacy were associated with implementing smoking cessation interventions [16]. In addition, the authors highlighted that support and assistance are needed for nurses [16].

In Croatia, no continuous interventions are aimed at patients to stop smoking [5]. Furthermore, we could not find any studies in the literature about knowledge, attitudes and implementation of smoking cessation interventions among nurses in Croatia. Having such knowledge could help support nurses in implementing smoking cessation interventions in Croatia.

Thus, this study aimed to examine which interventions for smoking cessation are carried out by nurses in Croatia and to examine their knowledge and attitudes about these interventions.

## Methods

### Study design

This was a cross-sectional study.

### Ethics

The study was conducted in accordance with the institutional Codes of Ethics. All methods were performed in accordance with the relevant guidelines and regulations. Written informed consent was obtained from all study participants.

The study protocol was approved by the Ethics Committee of the Sestre Milosrdnice University Hospital Center (Document number: Klasa 003–06/22-03-003; Urbroj: 251-29-11-22-01-9).

### Participants

The participants were nurses employed at the Sestre Milosrdnice University Hospital Center at the Department of Internal Medicine, the Department of Oncology and Nuclear Medicine, the Department of Cardiovascular Diseases, the Department of Neurology, the Department of Neurosurgery, the Department of Surgery, the Department of Psychiatry and the University Hospital for Tumors. Those departments were chosen as their patients are hospitalized longer than in other departments, so there are more opportunities for healthcare workers to implement interventions for smoking cessation.

### Data collection and study tool

In agreement with the head nurses of the departments, the participants were approached at their workplaces. Participation in the study was voluntary and anonymous. The study was conducted via a questionnaire from April to June 2022 (Appendix 1). The participants were approached in person in their workplace by author Lovro Ackar. Since the survey was conducted in early 2022, during the COVID-19 pandemic, there were still heavy restrictions in the hospital to the in-person access to nurses, and thus not all nurses that worked in the targeted departments were invited. We invited a convenience sample of nurses that we could contact in person. The participants received written information about the study, informed consent and the survey, and the kind request to participate. Nurses filled out the questionnaire in their department after reading the study information and signing the written informed consent. Surveys were filled out in paper form.

The first part of the questionnaire contained five questions about the sociodemographic data of the participants, including sex, professional education, smoking status, the department where they work and years of nursing experience. The second part of the questionnaire included the Helping Smokers Quit (HSQ) survey, developed and validated by Sarna et al. [17]. We obtained permission from Prof. Linda Sarna to use the questionnaire. The questionnaire consists of questions related to the frequency of implementation of the 5 A (Ask, Advise, Assess, Assist, Arrange) intervention for smoking cessation in addition to items about referring to a telephone quitline for cessation support, recommending tobacco cessation medications, reviewing barriers to quitting, and recommending a smoke-free home. Nurses assessed the frequency using one of five responses, including “always“, “usually“, “sometimes“, “rarely“, or “never“.

Furthermore, the questionnaire contained questions about the smoking status of nurses and their education about smoking cessation. At the end of the questionnaire, items about their attitudes toward smoking cessation interventions and perceived skills for implementing such interventions were scored with a 5-item Likert scale ranging from “completely disagree” to “completely agree” [18, 19] (Appendix 1).

The questionnaire was delivered in the Croatian language. For the study, the questions were translated into Croatian from English. Before delivering the survey to the nurses, the questionnaire was tested on a sample of nursing students employed in the healthcare system who were not part of the study sample. The pilot testing was used to assess the readability and understandability of the questionnaire. Following the pilot testing, there were no revisions to the questionnaire.

### Statistical analysis

Data were analyzed using descriptive statistics, including frequencies, percentages, medians and interquartile range. A simple analysis of variance was used to compare the average number of respondents. We compared the association between the implementation of smoking cessation interventions among nurses who smoke and those who do not. We did not use any imputation methods. The value P < 0.05 was used as the level of significance. The SPSS software (version 20, IBM, New York, USA) was used for statistical analysis.

## Results

There were 824 nurses employed in the targeted departments. However, due to COVID-19 restrictions on access to nurses, 340 questionnaires were distributed to nurses; 258 were filled out and returned (response rate: 31%). Raw data collected within this study are included in Appendix 2, which accompanies the manuscript.

The median age of participants was 36 years (interquartile range: from 26 to 48 years). The participants had a median of 12 years of nursing experience (interquartile range: 4 to 12 years). Most participants (88%) were women. Most participants were employed at the Department of Cardiovascular Diseases and the Department of Surgery. Most nurses completed Bachelor’s degree in nursing as the highest educational degree (Table 1).

**Table 1:** Characteristics of participants in terms of sex, education and department of employment

Variable		N	%*
Sex	Man	32	12
	Woman	226	88
Highest education level	Nursing high school	110	43
	Bachelor’s degree in nursing	116	45
	Master’s degree in nursing	31	12
	PhD degree	0	0.0
Department of employment	Department of Internal Medicine	50	19
	Department of Oncology and Nuclear Medicine	11	4
	Department of Cardiovascular Diseases	53	21
	Department of Neurology	13	5.0
	Department of Neurosurgery	5	1.9
	Department of Surgery	54	21
	Department of Psychiatry	25	9.7
	University Hospital for Tumors	47	18

*The percentages may not add up to exactly 100% due to rounding

Among the participants, 44% (N = 110) were smokers, and 9.6% (N = 11) smoked within five minutes after waking up. More than half of nurses smoked daily (54%; N = 139). One hundred twenty-two participants responded to a question about whether they tried to stop smoking within the past year; 39% (N = 48) indicated they did. When asked if they were trying to quit smoking currently, 147 participants responded, and 15% (N = 22) indicated that they did (Table 2). The median age when they started smoking was 18 years (interquartile range: 16 to 20 years). For those who stopped smoking, the median age when they stopped smoking was 31 years (interquartile range: 24 to 40 years).

**Table 2:** Smoking status of the participants

Question		N	%*
Have you ever smoked 100 or more cigarettes in your life?	Yes	139	54
	No	117	46
Do you smoke now?	Yes	110	44
	No	140	56
Do you smoke every day?	Yes	100	54
	No	87	47
How soon after you wake up do you smoke your first cigarette?	Within 5 min	11	9.6
	6–30 min	38	33
	31–60 min	29	25
	> 60 min	37	32
During the past 12 months, did you made a serious attempt to quit smoking (not smoking for 24 h or more)?	Yes	48	39
	No	74	61
Over your *lifetime*, how many times have you made a serious attempt to quit smoking (not smoking for 24 h or more)?	0 to1	53	43
	2 to 4	51	41
	5 to10	9	7.3
	> 10	11	8.9
Are you currently trying to quit?	Yes	22	15
	No	125	85

*The percentages may not add up to exactly 100% due to rounding

When asked about their training, 82% (214) of participants indicated that they did not participate in any smoking cessation education during their training. Furthermore, 96% (N = 243) of participants indicated that they did not take part in any education regarding smoking cessation within the past 24 months.

There were 61% (N = 159) of nurses who indicated that they always or usually ask patients about their smoking status; 29% (N = 75) always or usually advise them to stop smoking; 24% (N = 61) assess readiness to quit smoking always or usually. There were 13% (N = 35) of nurses who indicated they always or usually assist with smoking cessation; 8% (N = 20) always or usually arrange smoking cessation follow-up; 5% (N = 13) always or usually recommend the telephone smoking quitline. There were 12% (N = 31) of nurses who indicated they always or usually refer patients to community cessation resources; 5% (N = 13) recommend always or usually tobacco cessation medications; 10% (N = 24) review barriers to quitting always or usually, and 13% recommend patients to create a smoke-free environment (Table 3).

**Table 3:** Frequency of nurses’ delivery of smoking cessation interventions

Delivery of 5As and Other Interventions	N	%*
**Ask about smoking/tobacco use**		
Always	112	43
Usually	47	18
Sometimes	56	22
Rarely	31	12
Never	10	3.9
**Advise patients to quit smoking**		
Always	26	10
Usually	49	19
Sometimes	85	33
Rarely	63	25
Never	33	13
**Assess readiness to quit smoking**		
Always	16	6.3
Usually	45	18
Sometimes	78	31
Rarely	84	33
Never	32	13
**Assist with smoking cessation**		
Always	7	2.7
Usually	28	11
Sometimes	75	29
Rarely	85	33
Never	60	24
**Arrange smoking cessation follow-up**		
Always	3	1.2
Usually	17	6.7
Sometimes	38	15
Rarely	75	29
Never	122	48
**Recommend the telephone quitline**		
Always	3	1.2
Usually	10	3.9
Sometimes	30	12
Rarely	51	20
Never	161	63
**Refer to community cessation resources**		
Always	8	3.1
Usually	23	9.0
Sometimes	39	15
Rarely	69	27
Never	116	46
**Recommend tobacco cessation medications**		
Always	2	0.8
Usually	11	4.3
Sometimes	32	13
Rarely	64	25
Never	146	57
**Review barriers to quitting**		
Always	2	0.8
Usually	22	8.7
Sometimes	57	22
Rarely	76	30
Never	97	38
**Recommend creating a smoke-free home environment**		
Always	14	5.5
Usually	19	7.5
Sometimes	64	25
Rarely	61	24
Never	97	38

*The percentages may not add up to exactly 100% due to rounding

Responses to items regarding nurses’ attitudes about helping patients quit smoking revealed that most participants chose neutral responses, i.e., they neither agreed nor disagreed with the items (Table 4). There were 24% (N = 62) of participants who agreed or strongly agreed that asking patients about smoking increases the likelihood that they will quit. 54% (N = 138) of participants agreed or strongly agreed that it is difficult for them to get people to quit smoking. 24% (N = 62) agreed or strongly agreed that counseling patients about quitting is not an efficient use of their time. 20% (N = 50) agreed or strongly agreed that patients appreciate it when they provide advice about quitting smoking. Only 13% (N = 33) agreed or strongly agreed that discussing smoking cessation improves their relationship with patients, while 15% (N = 46) agreed or strongly agreed that they feel uncomfortable asking patients whether they smoke (Table 4).

**Table 4:** Nurses’ Attitudes About Helping Patients to Quit Smoking

Item	N	%*
**Asking patients about smoking increases the likelihood that they will quit.**		
Strongly disagree	25	9.8
Disagree	63	25
Neutral	104	41
Agree	51	20
Strongly agree	11	4.3
**It is difficult for me to get people to quit smoking.**		
Strongly disagree	6	2.4
Disagree	16	6.3
Neutral	94	37
Agree	105	41
Strongly agree	33	13
**Counseling patients about quitting is not an efficient use of my time**		
Strongly disagree	13	5.1
Disagree	79	31
Neutral	100	39
Agree	44	17
Strongly agree	18	7.1
**Patients appreciate it when I provide advice about quitting smoking.**		
Strongly disagree	20	7.9
Disagree	62	24
Neutral	122	48
Agree	40	16
Strongly agree	10	3.9
**Discussing smoking cessation improves my relationship with patients.**		
Strongly disagree	32	13
Disagree	74	29
Neutral	115	45
Agree	27	11
Strongly agree	6	2.4
**I feel uncomfortable asking patients whether they smoke.**		
Strongly disagree	41	16
Disagree	92	36
Neutral	77	30
Agree	38	15
Strongly agree	8	3.1
**As a nurse, I can play an important role in helping patients quit.**		
Strongly disagree	9	3.5
Disagree	31	12
Neutral	99	39
Agree	91	36
Strongly agree	24	9.4
**I need more training to help patients quit smoking.**		
Strongly disagree	20	7.9
Disagree	54	21
Neutral	84	33
Agree	68	27
Strongly agree	28	11
**I have insufficient time to counsel patients about quitting smoking.**		
Strongly disagree	9	3.5
Disagree	32	13
Neutral	77	30
Agree	89	35
Strongly agree	47	19
**I should take a more active role in helping patients to quit smoking.**		
Strongly disagree	13	5.1
Disagree	46	18
Neutral	112	44
Agree	66	26
Strongly agree	17	6.7
**Patients will be offended if I inquire about their smoking status.**		
Strongly disagree	17	6.7
Disagree	76	30
Neutral	113	45
Agree	41	16
Strongly agree	7	2.8
**Providing tobacco cessation counseling is important to our hospital even if only a few patients quit.**		
Strongly disagree	8	3.1
Disagree	18	7.1
Neutral	100	39
Agree	99	39
Strongly agree	29	11.4
**I have an obligation to advise patients on the health risks associated with tobacco use.**		
Strongly disagree	9	3.5
Disagree	28	11
Neutral	98	39
Agree	89	35
Strongly agree	30	12
**How many patients do you estimate have you counseled for smoking cessation** ***over the past week*** **?**		
0	158	62
1–2	71	28
3–5	15	5.9
More than 5	12	4.7

*The percentages may not add up to exactly 100% due to rounding

46% (N = 115) of participants agreed or strongly agreed that, as a nurse, they could play an important role in helping patients quit. 38% (N = 96) agreed or strongly agreed they need more training to help patients quit smoking. Among participants, 54% (N = 136) agreed or strongly agreed they have insufficient time to counsel patients about quitting smoking. 33% (N = 83) agreed or strongly agreed they should take a more active role in helping patients to quit smoking. 19% (N = 48) of participants agreed or strongly agreed that patients would be offended if they inquired about their smoking status (Table 4).

Half (N = 128) of the participants agreed that providing tobacco cessation counseling is important to the hospital, even if only a few patients quit. There were 47% (N = 119) of nurses who agreed or strongly agreed with a statement that they have an obligation to advise patients on the health risks associated with tobacco use (Table 4). However, very few participants, only 4.7% (N = 12), indicated that over the past week, they counselled more than five patients about smoking cessation (Table 4).

When they had to score statements about nurses and tobacco control, most nurses chose neutral responses (Table 5). 47% (N = 119) of participants agreed that nurses should set a good example by not smoking, 48% (N = 122) agreed that nurses should be involved in actively helping patients to stop smoking, and 48% (N = 121) agreed that nurses need additional training/skills in tobacco control (Table 5).

**Table 5:** Participants’ responses regarding statements about nurses and tobacco control

Items	N	%*
**Nurses should set a good example by not smoking**		
Strongly disagree	28	11
Disagree	30	12
Neutral	75	30
Agree	74	29
Strongly agree	45	18
**Nurses should be involved in actively helping patients to stop smoking**		
Strongly disagree	12	4.7
Disagree	20	7.9
Neutral	100	39
Agree	92	36
Strongly agree	30	12
**Nurses need additional training/skills in tobacco control**		
Strongly disagree	19	7.5
Disagree	22	8.7
Neutral	90	36
Agree	78	31
Strongly agree	43	17

*The percentages may not add up to exactly 100% due to rounding

At the end of the questionnaire, participants were asked to rate two questions with a Likert scale ranging from 1 (least important) to 5 (most important). Most participants chose a neutral response regarding the importance of nurses’ involvement in tobacco control activities (37%; N = 96). Also, when asked to assess how important it is for nurses to be involved in tobacco control activities, compared to other disease prevention activities (e.g., nutrition, exercise, etc.), most participants chose the neutral response (38%; N = 97).

Analysis of variance was conducted to analyze the association of items regarding nursing interventions for smoking cessation and nurses’ smoking status (Table 6). Among all questions and items given to the participants, a significant difference in the frequency of responses was noted for the item on reviewing barriers to quitting (P = 0.010) – nurses who smoke were engaged in such behavior significantly less than those that do not smoke. Also, we found a significant difference in the responses to the statement “Nurses should set a good example by not smoking“– agreement with this statement was more common among nurses who did not smoke compared to those who smoke (P < 0.001) (Table 6).

**Table 6:** Distribution of participants with regard to personal smoking status and questions/items regarding smoking cessation interventions implemented by nurses and their attitudes towards those interventions

		Do you smoke now?				P
		Yes		No		
		N	%*	N	%*	
**Ask about smoking/tobacco use**	Always	55	50	56	40	0.167
	Usually	13	12	31	22	
	Sometimes	27	25	28	20	
	Rarely	12	11	19	14	
	Never	3	2.7	6	4.3	
**Advise patients to quit smoking**	Always	8	7.3	16	12	0.078
	Usually	17	16	31	22	
	Sometimes	34	31	48	35	
	Rarely	30	27	32	23	
	Never	21	19	12	8.6	
**Assess readiness to quit smoking**	Always	3	2.7	12	8.6	0.276
	Usually	21	19	23	17	
	Sometimes	32	29	45	32	
	Rarely	37	33	44	32	
	Never	17	16	15	11	
**Assist with smoking cessation**	Always	3	2.7	4	2.9	0.682
	Usually	11	10	16	12	
	Sometimes	34	31	38	27	
	Rarely	32	29	51	37	
	Never	30	27	30	22	
**Arrange smoking cessation follow-up**	Always	2	1.8	1	0.7	0.851
	Usually	6	5.5	11	7.9	
	Sometimes	17	16	19	14	
	Rarely	33	30	40	29	
	Never	52	47	68	49	
**Recommend the telephone quitline**	Always	2	1.8	1	0.7	0.442
	Usually	4	3.6	6	4.3	
	Sometimes	17	16	12	8.6	
	Rarely	20	18	31	22	
	Never	67	61	89	64	
**Refer to community cessation resources**	Always	4	3.6	4	2.9	0.312
	Usually	9	8.2	14	10	
	Sometimes	15	14	23	17	
	Rarely	25	23	44	32	
	Never	57	52	54	39	
**Recommend tobacco cessation medications**	Always	1	0.9	1	0.7	0.665
	Usually	4	3.6	7	5.0	
	Sometimes	13	12	17	12	
	Rarely	23	21	39	28	
	Never	69	63	75	54	
**Review barriers to quitting**	Always	2	1.8	0	0.0	0.010
	Usually	6	5.5	16	12	
	Sometimes	21	19	32	23	
	Rarely	26	24	48	35	
	Never	54	50	43	31	
**Recommend creating a smoke-free home environment**	Always	5	4.5	9	6.5	0.166
	Usually	4	3.6	15	11	
	Sometimes	29	26	33	24	
	Rarely	25	23	36	26	
	Never	47	43	46	31	
**Asking patients about smoking increases the likelihood that they will quit.**	Strongly disagree	14	13	10	7.2	0.178
	Disagree	32	29	29	21	
	Neutral	42	39	61	44	
	Agree	18	17	32	23	
	Strongly agree	3	2.8	7	5.0	
**It is difficult for me to get people to quit smoking.**	Strongly disagree	2	1.8	4	2.9	0.451
	Disagree	4	3.7	11	7.9	
	Neutral	41	38	50	36	
	Agree	45	41	60	43	
	Strongly agree	17	16	14	10	
**Counseling patients about quitting is not an efficient use of my time**	Strongly disagree	5	4.6	6	4.3	0.293
	Disagree	28	26	51	37	
	Neutral	48	44	50	36	
	Agree	18	17	25	18	
	Strongly agree	10	9.2	7	5.0	
**Patients appreciate it when I provide advice about quitting smoking.**	Strongly disagree	12	11	8	5.8	0.170
	Disagree	26	24	34	25	
	Neutral	51	47	69	50	
	Agree	13	12	25	18	
	Strongly agree	7	6.4	3	2.2	
**Discussing smoking cessation improves my relationship with patients.**	Strongly disagree	17	16	14	10	0.594
	Disagree	28	26	46	33	
	Neutral	50	46	61	44	
	Agree	11	10	15	11	
	Strongly agree	3	2.8	3	2.2	
**I feel uncomfortable asking patients whether they smoke.**	Strongly disagree	17	16	21	15	0.526
	Disagree	37	34	54	39	
	Neutral	31	28	44	32	
	Agree	21	19	16	12	
	Strongly agree	4	3.6	4	2.9	
**As a nurse, I can play an important role in helping patients quit.**	Strongly disagree	6	5.5	3	2.2	0.633
	Disagree	11	10	18	13	
	Neutral	44	40	54	39	
	Agree	39	36	50	36	
	Strongly agree	9	8.3	14	10	
**I need more training to help patients quit smoking.**	Strongly disagree	10	9.2	8	5.8	0.715
	Disagree	22	20	32	23	
	Neutral	34	31	48	35	
	Agree	29	27	38	27	
	Strongly agree	14	12.8	13	9.4	
**I have insufficient time to counsel patients about quitting smoking.**	Strongly disagree	5	4.6	4	2.9	0.943
	Disagree	13	12	16	12	
	Neutral	34	31	42	30	
	Agree	36	33	51	37	
	Strongly agree	21	19	26	19	
**I should take a more active role in helping patients to quit smoking.**	Strongly disagree	6	5.5	7	5.0	0.985
	Disagree	19	17	25	18	
	Neutral	46	42	63	45	
	Agree	30	28	35	25	
	Strongly agree	8	7.3	9	6.5	
**Patients will be offended if I inquire about their smoking status.**	Strongly disagree	9	8.3	8	5.8	0.494
	Disagree	26	24	47	34	
	Neutral	51	47	60	43	
	Agree	20	18	20	14	
	Strongly agree	3	2.8	4	2.9	
**Providing tobacco cessation counseling is important to our hospital even if only a few patients quit.**	Strongly disagree	3	2.8	5	3.6	0.732
	Disagree	8	7.3	9	6.5	
	Neutral	45	41	53	38	
	Agree	44	40	53	38	
	Strongly agree	9	8.3	19	14	
**I have an obligation to advise patients on the health risks associated with tobacco use.**	Strongly disagree	6	5.5	3	2.2	0.315
	Disagree	14	13	14	10	
	Neutral	46	42	51	37	
	Agree	33	30	52	37	
	Strongly agree	10	9.2	19	13.7	
**How many patients do you estimate have you counseled for smoking cessation** ***over the past week*** **?**	0	75	68	79	57	0.160
	1 to 2	25	23	45	32	
	3 to 5	4	3.6	10	7.2	
	More than 5	6	5.5	5	3.6	
**Nurses should set a good example by not smoking**	Strongly disagree	18	17	10	7.2	< 0.001
	Disagree	19	18	11	8.0	
	Neutral	27	25	47	34.1	
	Agree	35	32	37	26.8	
	Strongly agree	9	8.3	33	24	
**Nurses should be involved in actively helping patients to stop smoking**	Strongly disagree	9	8.3	3	2.2	0.118
	Disagree	11	10	9	6.5	
	Neutral	41	378	55	40	
	Agree	39	36	52	38	
	Strongly agree	9	8.3	19	14	
**Nurses need additional training/skills in tobacco control**	Strongly disagree	11	10	7	5.1	0.235
	Disagree	10	9.3	11	8.0	
	Neutral	36	33	53	38	
	Agree	38	35	40	29	
	Strongly agree	13	12	27	20	
**How important is it for nurses to be involved in tobacco control activities?**	1 – least important	9	8.3	14	10	0.372
	2	23	21	17	12	
	3	43	39	53	38	
	4	27	25	42	30	
	5 – the most important	7	6.4	12	8.7	
**Compared to other disease prevention activities (e.g., nutrition, exercise, etc.), how important is it for nurses to be involved in tobacco control activities?**	1 – least important	14	13	11	8.0	0.475
	2	21	19	19	14	
	3	39	36	58	42	
	4	28	26	39	28	
	5 – the most important	7	6.4	11	8.0	

*The percentages may not add up to exactly 100% due to rounding

## Discussion

We found that almost half of the nurses (44%) who participated in the study were smokers. Slightly less than half of the respondents (43%) always ask the patient if they use tobacco products, thus opening up the possibility of using interventions to stop smoking. Very few respondents indicated that they always help the patient to quit smoking. Almost all respondents did not receive any education in the past two years that would have provided them with the knowledge and skills to assist in quitting smoking, and most never had such education while they were attending nursing school. Few differences in the use of nursing interventions and attitudes towards those interventions were observed between nurses who smoked versus those who did not.

According to US Centers for Disease Control data, 25% of nurses worldwide consume tobacco products. Studies conducted during the COVID-19 pandemic indicated that a high percentage of nurses consumed tobacco, alcohol or other substances due to anxiety, stress and the effect of the pandemic [20, 21]. In 2019, Nilan et al. published a systematic review and meta-analysis of the prevalence of tobacco use in healthcare workers. They included 229 studies representing 457,415 healthcare workers from 63 countries. They found that the overall pooled prevalence of tobacco use in healthcare workers was 21%, with 31% in men and 17% in women. The highest estimates for the overall pooled prevalence of tobacco use were found for men physicians and women nurses. The percentage of women nurses smoking in high-income countries was 21%, and in upper-middle-income countries, it was 25% [22].

Compared to these countries, the percentage of smokers in this study was very high, as 44% of nurses in our sample indicated they were smokers. Furthermore, almost 10% of them have their first cigarette within five minutes of waking up. This finding indicates that many nurses probably have a problem with nicotine addiction since the time of the first cigarette upon waking is one of the indicators of the heaviness of smoking or high nicotine addiction [23].

A nurse should be sufficiently educated, eloquent, professional, kind, and must possess many skills to provide high-quality healthcare. Nursing interventions also include counseling and the promotion of a healthier lifestyle. For example, in this study, almost half of the nurses stated that they always ask patients about their smoking status. The same distribution of responses was found in a study from the Czech Republic conducted on a sample of 157 nurses [13].

We found that most nurses were not ready to help the patient quit smoking, and the question is whether they are even aware of the importance of their role in this. Research on this topic has been conducted in other countries as well. The same problem was encountered with registered nurses in China; most indicated that they asked patients about their smoking status, but few were willing to help with smoking cessation interventions [24].

In our sample, most nurses never advised patients to use the smoking cessation hotline. It is unclear whether the nurses are aware of the various existing resources for smoking cessation in the community. Also, most nurses rarely or never recommend patients’ treatment in the community (clinics, counseling centers, etc.), and most do not recommend medicines that help in smoking cessation. A study conducted by Sarna et al. in the Czech Republic showed that slightly less than 15% of nurses always recommend community treatment compared to surveyed nurses in Croatia, who always recommend community treatment in less than 3% [13].

Sarna et al. concluded that their data demonstrate that consistent nursing intervention to help patients quit smoking in the Czech Republic is relatively rare. Also, some nurses were reluctant to intervene with patients on this topic [13].

As for the nurses’ attitudes, they were mostly neutral about most of the statements about nurses and smoking cessation interventions. This could be seen as taking the “line of least resistance” and against making additional effort within their working framework. A third of nurses indicated that patients would not change their smoking habits if asked about their tobacco product consumption habits. More than 40% considered persuading the patient to quit smoking difficult. Such reasoning is likely due to the lack of nurses’ self-confidence and professional limitations since less than 10% of nurses agreed that they could persuade a patient to stop smoking. Professional self-confidence is a part of professional identity, competence, clinical knowledge and reasoning that could be lacking in this field [25].

Many nurses were aware that their role in smoking prevention is important, as 36% of them agreed that counseling on quitting is not a waste of time – this was in contrast to the 24% of nurses who agreed that counseling is a waste of time. However, less than a fifth of them indicated that patients appreciate being advised, and almost half indicated that discussing smoking will not lead to a better relationship with the patient. This is far from the recommendations of professionals who advise smoking cessation as part of health promotion. Health promotion is achieved by advocating the basic prerequisites for a healthy life, enabling the patient’s health potential and mediating between patients and their social interests [26].

Participants’ responses regarding their education about smoking cessation could be a reason for this inactivity. More than a third of respondents agreed that they need additional education regarding smoking cessation and that they should become active in the field of patient education and counseling. This is essential information that should be considered if programs for nurses were to be built on the control of tobacco products and patient counseling so that they could be empowered to engage in interventions that could help patients in smoking cessation. In addition, other research results strongly support the need for additional education for nurses regarding smoking cessation [13].

Few participants in our study received education on smoking cessation, and almost half of the respondents indicated they needed education on quitting smoking. Those results align with many prior studies that showed nurses report a lack of education regarding smoking cessation [7, 13, 27–29].

We found very few differences in behaviors and attitudes of nursing regarding interventions for smoking cessation. In a review regarding the smoking habits of nurses and interventions towards patients between 1996 and 2015, Duaso et al. showed that the personal smoking status of nurses is not significantly related to nurses questioning patients about their smoking. It was also confirmed that nurses who smoke were 13% less likely to advise their patients to stop smoking and 25% less likely to arrange to stop smoking afterwards [30].

The feasibility of the multimodal intervention for smoking cessation in a hospital setting in Croatia was proven in a recently published pilot study. Patients were very satisfied with the intervention, and high smoking abstinence rates were recorded at six-month after hospital discharge. Thus, healthcare workers employed in hospitals could be educated to provide such interventions regularly [31].

Nurses should engage in interventions for smoking cessation. Patients trust nurses the most among healthcare professionals; thus, they could play an important role in the smoking cessation effort among patients. Unfortunately, this research has shown that only some nurses engage in interventions that could help patients quit smoking. It is acknowledged that nurses may be overloaded with their other duties. However, if they became aware of the importance of participating in smoking cessation, they could help the patient and contribute to greater personal and work satisfaction.

Nurses’ education on smoking cessation counseling could potentially reduce the number of smokers since nurses are the most numerous workforce in Croatia, and properly focused education of nurses about helping patients with smoking cessation would certainly have an impact [32].

A limitation of our study is that we have included only one hospital in Croatia. However, the study was conducted during the COVID-19 pandemic when personal contact with healthcare professionals in hospitals was extremely limited. Future studies in this area should explore interventions for helping nurses quit smoking. Also, researchers should test the efficacy of various interventions that will help nurses change their attitudes towards nursing smoking cessation interventions and foster their engagement in such interventions. Studies reporting the design and evaluation of such interventions and their acceptability to nurses will help us design and implement successful interventions in the future. After adopting such interventions, future follow-up studies should explore nurses’ attitudes, experiences and implementation of smoking cessation practices. In that respect, our study can serve as a benchmark, reporting the baseline data that can serve as a comparison for future studies in Croatia on this topic.

Of note, there were missing data in several surveys, as not all participants answered all of the questions. The percentage of missing answers in any given question was at most 5%.

## Conclusion

Our results point to the importance of educating nurses about smoking cessation interventions they could engage in. Furthermore, our results point to the need for introducing courses about smoking cessation during the nurses’ compulsory education, with which they would acquire competencies for implementing smoking cessation interventions in the workplace. Likewise, the high prevalence of smoking among nurses indicates the need to develop smoking cessation interventions targeting nurses and other healthcare workers who smoke.

## Electronic supplementary material

Below is the link to the electronic supplementary material.

Supplementary Material 1

Supplementary Material 2

## Data Availability

Raw data collected within this study are included in Appendix 2, which accompanies the manuscript.

## Abbreviations

5A: Ask, Advise, Assess, Assist, Arrange
ABC method: Ask, Brief advice, Cessation support
FCTC: Framework Convention on Tobacco Control
HSQ: Helping Smokers Quit
WHO: AWorld Health Organization
