# Supplementary material for: The knowledge, attitudes and behaviors of hospital nurses on smoking cessation interventions: a cross-sectional study
Source: BMC Nurs. 2023 Jul 3;22:228. doi: 10.1186/s12912-023-01394-7 (PMC10316570; doi:10.1186/s12912-023-01394-7)
Supplement: Supplementary file 1 — Supplementary Material 1 [file 12912_2023_1394_MOESM1_ESM.pdf]

Appendix 1. Questionnaire used in the study

Personal characteristics

1. **The month and year of your birth**: ____________________(month/year)

2. **What is your sex?** ☐ Male ☐ Female

Professional characteristics

3. What is the highest level of education in nursing you have completed? Please check one.

☐ Nursing High School
☐ Bachelor Degree
☐ Master's Degree
☐ PhD degree

4. How many years have you practiced as a nurse? _____________

5. Please write the name of your department:
____

Nursing interventions in the delivery of tobacco cessation interventions to patients.

6. **Please check the one box that most closely relates to your care of adult patients, using the scale below**.

**When you care for patients how often do you:**	**always**	**usually**	**sometimes**	**rarely**	**never**
Ask about a patient's smoking/ tobacco use?	☐	☐	☐	☐	☐

7. **Please check the one box that most closely relates to your care of adult patients, using the scale below**.

	**When you care for a <u>patient who smokes</u> how often do you:**	always	usually	sometimes	rarely	never
a.	Advise a patient to quit smoking?	☐	☐	☐	☐	☐
b.	Assess if patients are interested in stopping smoking?	☐	☐	☐	☐	☐
c.	Assist a patient with smoking cessation?	☐	☐	☐	☐	☐
d.	Arrange smoking cessation follow-up?	☐	☐	☐	☐	☐
e.	Recommend the use of a *telephone quitline* for smoking	☐	☐	☐	☐	☐
f.	Refer a patient to tobacco cessation resources (clinics, counseling, etc.) in the community?	☐	☐	☐	☐	☐
g.	Provide recommendations for tobacco cessation medications?	☐	☐	☐	☐	☐
h.	Review barriers to quitting with patients who are unwilling to make a quit attempt?	☐	☐	☐	☐	☐
i.	Recommend to patients and family members the importance of creating a smoke-free home environment after leaving the hospital?	☐	☐	☐	☐	☐

OPINIONS about COUNSELING PATIENTS to QUIT SMOKING

8. **Please rate the extent to which you agree with the following statements.**

CHECK ONLY ONE BOX		STRONGLY DISAGREE	DISAGREE	NEUTRAL	AGREE	STRONGLY AGREE
a.	Asking patients about smoking increases the likelihood that they will quit.	☐1	☐2	☐3	☐4	☐5
b.	It is difficult for me to get people to quit smoking.	☐1	☐2	☐3	☐4	☐5
c.	Counseling patients about quitting is not an efficient use of my time.	☐1	☐2	☐3	☐4	☐5
d.	Patients appreciate it when I provide advice about quitting smoking.	☐1	☐2	☐3	☐4	☐5
e.	Discussing smoking cessation improves my relationship with patients.	☐1	☐2	☐3	☐4	☐5
f.	I feel uncomfortable asking patients whether they smoke.	☐1	☐2	☐3	☐4	☐5
g.	As a nurse, I can play an important role in helping patients quit.	☐1	☐2	☐3	☐4	☐5
h.	I need more training to help patients quit smoking.	☐1	☐2	☐3	☐4	☐5
i.	I have insufficient time to counsel patients about quitting smoking.	☐1	☐2	☐3	☐4	☐5
j.	I should take a more active role in helping patients to quit smoking.	☐1	☐2	☐3	☐4	☐5
k.	Patients will be offended if I inquire about their smoking status.	☐1	☐2	☐3	☐4	☐5
l.	Providing tobacco cessation counseling is important to our hospital even if only a few patients quit.	☐1	☐2	☐3	☐4	☐5
m.	I have an obligation to advise patients on the health risks associated with tobacco use.	☐1	☐2	☐3	☐4	☐5

9. How many patients do you estimate have you counseled for smoking cessation ***over the past week?***

	None	1-2	3-5	More than 5
Patients counseled	☐	☐	☐	☐

Personal Smoking History

10. **Have you ever smoked 100 or more cigarettes in your life?**
(CHECK ONLY ONE BOX)
☐ Yes
☐ No
[if no, skip to question 26]

If yes:
11. **At what age did you start smoking?** ___________years.

12. **Do you smoke now?**
☐ Yes
☐ No
[if no, skip to question 26]

If yes:
13. **Do you smoke every day?**
☐ Yes
☐ No

If No:
14. **At what age did you quit smoking?** ______________years

---

15. **How soon after you wake up do you smoke your first cigarette? (Please check only one box)**
☐ Within 5 minutes
☐ 6 – 30 minutes
☐ 31-60 minutes
☐ > 60 minutes

16. **During the past 12 months, did you made a serious attempt to quit smoking (not smoking for 24 hours or more)?**
☐ Yes
☐ No

17. Over your ***lifetime,*** how many times have you made a serious attempt to quit smoking (not smoking for 24 hrs or more)?

Number of times:
- ☐ 0-1
- ☐ 2-4
- ☐ 5-10
- ☐ > 10

18. **Are you currently trying to quit?**
- ☐ Yes
- ☐ No

Tobacco cessation education

19. **Have you received training in smoking cessation <u>within the past 24 months</u>?**
- ☐ Yes
- ☐ No

20. **During your education, have you ever received any training related to smoking cessation counseling?**
- ☐ Yes
- ☐ No

Attitudes and beliefs about nurses and smoking

Please read each statement below and place a checkmark in the box that best represents how you feel:

CHECK ONLY ONE BOX.

21.	**Please tell us how strongly you agree or disagree with the following statements about nurses and tobacco control:**	Strongly disagree	Disagree	Not sure	Agree	Strongly agree
A	Nurses should set a good example by not smoking	☐**1**	☐**2**	☐**3**	☐**4**	☐**5**
B	Nurses should be involved in actively helping patients to stop smoking	☐**1**	☐**2**	☐**3**	☐**4**	☐**5**
C	Nurses need additional training/skills in tobacco control	☐**1**	☐**2**	☐**3**	☐**4**	☐**5**

22.	**Please rate the importance of these statements.**	**Least important**				**Most important**
a.	How important is it for nurses to be involved in tobacco control activities?	☐**1**	☐**2**	☐**3**	☐**4**	☐**5**

b. Compared to other disease prevention activities (e.g., nutrition, exercise, etc.), how important is it for nurses to be involved in tobacco control activities?

☐**1** ☐**2** ☐**3** ☐**4** ☐**5**
